# microRNA Detection via Nanostructured Biochips for Early Cancer Diagnostics

**DOI:** 10.3390/ijms24097762

**Published:** 2023-04-24

**Authors:** Sara Martino, Chiara Tammaro, Gabriella Misso, Michela Falco, Marianna Scrima, Marco Bocchetti, Ilaria Rea, Luca De Stefano, Michele Caraglia

**Affiliations:** 1 Department of Precision Medicine, University of Campania “Luigi Vanvitelli”, 80138 Naples, Italy; sara.martino@unicampania.it (S.M.); chiara.tammaro@unicampania.it (C.T.); michela.falco@unicampania.it (M.F.); marco.bocchetti@unicampania.it (M.B.); michele.caraglia@unicampania.it (M.C.); 2 Unit of Naples, National Research Council, Institute of Applied Sciences and Intelligent Systems, 80138 Naples, Italy; ilaria.rea@na.isasi.cnr.it; 3 Laboratory of Molecular and Precision Oncology, Biogem Scarl, Institute of Genetic Research, 83031 Ariano Irpino, Italy; marianna.scrima@biogem.it

**Keywords:** microRNA, biosensor, nanoparticle, nanostructured material, biochip, optical detection, electrochemical detection

## Abstract

MicroRNA (miRNA) are constituted of approximately 22 nucleotides and play an important role in the regulation of many physiological functions and diseases. In the last 10 years, an increasing interest has been recorded in studying the expression profile of miRNAs in cancer. Real time-quantitative polymerase chain reaction (RT-qPCR), microarrays, and small RNA sequencing represent the gold standard techniques used in the last 30 years as detection methods. The advent of nanotechnology has allowed the fabrication of nanostructured biosensors which are widely exploited in the diagnostic field. Nanostructured biosensors offer many advantages: (i) their small size allows the construction of portable, wearable, and low-cost products; (ii) the large surface–volume ratio enables the loading of a great number of biorecognition elements (e.g., probes, receptors); and (iii) direct contact of the recognition element with the analyte increases the sensitivity and specificity inducing low limits of detection (LOD). In this review, the role of nanostructured biosensors in miRNA detection is explored, focusing on electrochemical and optical sensing. In particular, four types of nanomaterials (metallic nanoparticles, graphene oxide, quantum dots, and nanostructured polymers) are reported for both detection strategies with the aim to show their distinct properties and applications.

## 1. Introduction

MicroRNAs (miRNAs) are small noncoding RNAs (approximately 18–22 nucleotides in length) that work as gene expression epigenetic regulators [1]. They are involved in the control of many physiological functions and diseases through several mechanisms including apoptosis, differentiation, and proliferation [2,3,4]. Cancer is the second leading cause of death in western countries. Recent studies have focused on the identification of diagnostic and prognostic circulating biomarkers in cancer and the profiling of miRNAs is recently emerging as a potential source of cancer biomarkers [5]. In fact, miRNAs are expressed in all body fluids such as saliva, blood, and semen at very low concentrations (in the range attomoles to femtomoles) [6,7]. Therefore, miRNA detection strongly needs reliable techniques that offer high levels of specificity and selectivity. The standard detection methods that have been used during the last 30 years are represented by real-time quantitative polymerase chain reaction (RT-qPCR) and microarrays [8,9,10]. However, they are costly, time-consuming due to long steps for sample preparation, have low sensitivity owing to the lack of a transducer element, and error-prone due to the fair number of false positive results [11,12].

Nanotechnology is a promising alternative to the classic technique for miRNA identification and allows the fabrication of nanostructured biosensors which are widely exploited in the diagnostic field. In general, a biosensor is defined as a device formed by a recognition system and a transducer element, providing analytical information [13]. The possibility to perform fast-response diagnostic and prognostic tests is particularly useful for early diagnosis at the initial stages of the disease [14]. Several positive aspects that are provided by nanostructured biosensors are (i) their large surface area tovolume ratio that allows the immobilization of a biorecognition element responsible for the capture of the analyte, (ii) the presence of a transduction element that transforms the signal after the contact with the target molecule, (iii) the possibility to increase the sensitivity and specificity leading to low limits of detection (LOD), and (iv) the ability to build portable, wearable, and low-cost products [15].

A significant issue is represented by the low miRNA concentration in body fluids. To date, several strategies have emerged as novel tools able to potentiate the efficiency of biosensors for detecting miRNAs. Amplification techniques represent a solid approach able to extend miRNAs short sequence (≈18–22 nucleotides) to increase the concentration, and, as a consequence, enhance the specificity of the target binding and the signal transduced by the biosensor [16]. In the last years, electrochemical biosensors have drawn attention in the prognostic and diagnostic field due to their simplicity, low-cost, biocompatibility, ease of use, and quick results, consolidating their position as a practical method for miRNA detection. The possibility to modify electrodes using nanomaterials (NMs) by improving surface probe immobilization has provided a high level of sensitivity since they are able to detect the low concentrations of target analytes [17,18,19]. Optical biosensors are analytical tools that enable a quick response time and simple detection of specific biological and chemical species. Once the biorecognition element (i.e., an enzyme, an antibody, an oligonucleotide sequence, and so on) interacts with the target molecule, an optical signal is generated, providing analytical information about the concentration of the investigated substance. The sensing strategy can be classified as label-free and label-based [20]. Recently, optical biosensors have established a significant role in miRNA quantification since they have further confirmed their ability to reach low detection limits and have high sensitivity and specificity. The different properties of nanomaterials (NM) make them important in the fabrication of optical biosensors. Taking advantages of NMs optical features, optical biosensors have been considered as an effective alternative to conventional molecular methods for detecting tiny molecules such as miRNAs [21].

In this review, the role of nanostructured biosensors in miRNA detection is explored, focusing on electrochemical and optical sensing. In particular, four types of nanomaterials (metallic nanoparticles, graphene oxide, quantum dots, and nanostructured polymers) are reported for both detection strategies.

## 2. Role of miRNAs as Cancer Biomarkers and Gold Standard Techniques for Their Detection

miRNAs were discovered for the first time in 1993 in *C. elegans* [22]. To date, the last version of miRbase (v22), the most important microRNA database, accounts for 48,860 mature miRNAs from 271 organisms [23]. Many studies performed over the last 30 years have deepened their role, function, and biogenesis. miRNAs biogenesis starts from the pre-miRNA sequences, that are then transcribed by RNA polymerase II or III. Subsequently, Drosha ribonucleases process pri-miRNA to pre-miRNA into the nucleus; pre-miRNA is transported by the exportin-5-Ran-GTP system in the cytoplasm where Dicer processes pre-miRNA in the mature miR:miR. miRNAs negatively regulate protein translation of the target mRNAs, by binding them at the 3′ untranslated region (UTR) which can determine both inhibition of mRNA translation and inhibition of the polyadenylation of the mRNA with the consequent decrease of its half-life in mammalian cells [24]. During the last 10 years, an increasing interest has been recorded in studying miRNA expression profiles in cancer, which is the second leading cause of death in western countries [5]. The first report about miRNA involvement in tumor pathogenesis was made by Dr. Croce et al. in 2002 when they described how miR-15a and miR16-1 were crucially relevant in the development of chronic lymphocytic leukemia [25]. This study represented the milestone of a new branch of cancer research which aimed to elucidate the oncogenic or tumor suppressor role of the small noncoding RNAs, useful for both therapeutic and diagnostic application [26]. The first evidence of miRNAs detected in a biological fluid is dated 2008 when Chim et al. recorded the presence of placental miRNAs in maternal plasma [27]; in the same year Mitchell et al. found an upregulation of miR-141 level in prostate cancer patients in comparison to healthy donors. Moreover, in this study it was described that miRNAs were protected by the RNAses, commonly expressed in human blood [28]. The stability of miRNAs in body fluids is related to the lipidic or the proteic structures they are bound to. In detail, miRNAs can be released from cells in the extracellular compartment through the accumulation in multi-vesicular bodies that can, in turn, be either degraded by lysosomes or excreted as exosomes, depending upon the activity of the sphingomyelinase 2. Exosomes are small vesicles up to 150 nm formed by an external phospholipid bilayer and an internal hydrophilic core containing miRNAs and other nucleic and proteic factors. Alternatively, miRNAs can be secreted in complex with proteins such as argonaute-2 (Ago-2), or even with HDL [29,30]. The discovery of precise miRNA signatures for specific cancers would simplify and especially avoid the delay in cancer diagnosis that often represents the main cause of death for many tumors [31,32]. miRNome analysis, which consists of miRNA sequencing or microarray analysis of miRNAs extracted from clinical samples, is a common approach used for identifying miRNAs as cancer biomarkers [33,34]. The statistically relevant dysregulated miRNAs become good candidates for the discovery of novel cancer biomarkers. The last GLOBOCAN update ranked the most commonly diagnosed cancers including breast (11.7%), lung (11.4%), colorectal (10.0%), and others. Moreover, GLOBOCAN has predicted an increasing number of new cancers for 2040, which are estimated to achieve 28.4 million in the world [35]. This alarming prediction encourages the development of new early diagnostic techniques. Different miRNA signatures have been described to distinguish breast cancer with acceptable sensitivity and specificity, such as miR-25-3p, -29a-5p, -105-5p, 181b1-5p, -335-5p, and -339-5p [36], discovered to be dysregulated in cancer tissues. Moreover, a panel composed of miR-148b, -376c, -409-3p -801, -127-3p, -376a, and -652 were expressed at significantly higher levels in the plasma when compared to healthy women samples [37]. This category of biomarkers is not limited to diagnostic potential, but also to the prognostic one [38]. The above cited miRNA signatures are taken just as an example of thousands of papers describing the role of miRNAs as diagnostic or prognostic biomarkers in cancer. In the last few years, an increasing number of comprehensive tumor-specific biomarker reports have been published [7,39,40] and miRNAs are described as one of the most prominent candidates. However, despite the considerable amount of scientific work undertaken in this direction, this promising field of research was defined as a drop in the ocean by Nature journal in 2011 [41] because of the few numbers of miRNAs that are in clinical trials. The discrepancy between the numbers concerning the preclinical and clinical phases could be linked not only to the lack of funding for clinical studies, but also for the many issues surrounding the precise and fast quantification that have revealed more challenges than expected and, not least, for the normalization process [42]. These findings should address future scientific efforts in trying to increase the number of miRNA signature studies in the clinical phase to develop a new reliable class of biomarkers.

The huge interest for this class of small RNAs as predictive biomarkers in liquid biopsies clashes with a series of problems inherent in their nature. Firstly, they are just 0.01% of the whole RNAs and, for this reason, their concentration is very low [43]. Moreover, miRNAs coming from the same family present high homology in sequences, which evolves in overlapping messenger RNA targets or even distinct ones that often are mRNAs belonging to the same pathway [44]. The detection methods considered as gold standard techniques share the same starting point which is RNA extraction. The isolation step is usually performed by using a commercial kit, such as miRNeasy Serum/Plasma Kit^®^ (Qiagen), miRVana PARIS Kit^®^ (Ambion) etc., which provide high reliability and reproducibility and often combine the organic extraction with filter-based step. The quality control of the extracted RNA is then assessed using different instruments, such as Agilent 2100 Bioanalyzer^®^, Qubit^®^ microRNA assay, and the most used NanoDrop spectrophotometer^®^. After this mandatory step, different methods for detection can be performed, each one presenting specific advantages and disadvantages. 

### 2.1. Northern Blotting

The Northern blot is one of the most deep-rooted techniques in miRNA detection; the first miRNA discovered was indeed isolated with this method. The technique is a variant of the Southern blot, directed for DNA separation, where it ironically takes the name from [45]. The Northern blot is based on electrophoretic separation of nucleotide fragments which are then transferred to a solid support, and subsequently exposed to a DNA molecule of a known and marked sequence acting as a probe [46]. The amount of requested RNA is in the range of 2–5 µg and should be previously denatured at 75–85 °C for 5 min. The separated RNA is transferred on a positively charged nylon membrane by using a basic buffer. The RNA on the membrane is then fixed by a thermal process using UV radiation. Moreover, a more sensitive strategy for small RNA isolation has been reported which substitutes the UV step with a 1-ethyl-3-(3-dimethylaminopropyl) carbodiimide (EDC)-mediated chemical cross-linking step [8,47]. The third and last step is a hybridization of the fixed RNA with a labelled complementary strand probe in a specific buffer at a melting temperature of 37–42 °C. The Northern blot is a robust and run-in technique, but it requires a huge amount of RNA, it is time-consuming, poorly specific, and not quantitative. Due to these limitations, it is not frequently performed.

### 2.2. Real-Time Quantitative Polymerase Chain Reaction (RT-qPCR)

Real-time quantitative polymerase chain reaction (RT-qPCR) is currently the most widely used method, thanks to the high sensitivity [48], specificity shown at single base level [10], and the costs. Moreover, the analysis procedure is easy and well-established. The first step is the retro transcription from total RNA to cDNA (complementary DNA). The second step consists of cDNA amplification following its interception by PCR primers. However, because of the difficulty in distinguishing the sequences given the short miRNA length, a further step of sequence extension must be performed. There are two different approaches that can be used: (i) the RT-primers can extend the miRNA sequences during the cDNA synthesis or (ii) before the reverse transcription, the sequences are previously opportunely extended to be recognized by the universal RT-primers. Moreover, the design of the RT-specific primers can be either linear or containing a stem loop, while the universal RT-primers are directed to select the poly(A) tail, previously added to the sequences. The amplification that occurs during the PCR is quantitative for the use of dyes or probes, such as hydrolysis probes [49]. However, despite the many advantages discussed so far, the normalization process remains debated since it can be performed selecting an “internal control” or even adding a “spike in control”. 

### 2.3. Microarrays

Microarrays are a high throughput analysis system where miRNAs can be studied without an amplification procedure. For this reason, miRNAs are previously labelled, then exposed to probes, such as a stem loop probe or LNA, to obtain hybridization and, finally, a quantification of the signal [50]. This technique has been modified many times over the years to increase the sensitivity and the specificity which still represent a limitation [51]. In fact, this platform remains a standard technique directed to those studies where there is an interest for large testing of samples for already discovered miRNAs.

### 2.4. miRNA-seq or Small RNA-seq

Small RNA sequencing (RNA-Seq) is a technique to isolate and sequence small RNA species, such as microRNAs (miRNAs). Moreover, it allows the characterization of the variation such as isomiRs with single-base resolution, as well as the analysis of any small RNA or miRNA without prior sequence or secondary structure information. These systems are also called Next Generation Sequencing [52]. The major systems are the Illumina^®^ platform which supports TruSeq Small RNA Library Preparation Kits to prepare specific miRNA-Seq libraries and Ion Torrent technology based on the “sequencing by synthesis” method which outperforms pyrosequencing with respect to sensitivity. The first enables multiplexed sequencing with the introduction of 48 unique indexes, allowing miRNA and small RNA discovery and profiling throughput to match the unparalleled output of Illumina sequencing. Illumina-based sequencing technology represents a “reversible terminator sequencing” method [53]. SOLiD (sequencing by oligonucleotide ligation and detection) exhibits high accuracy as each base is sequenced twice, but the read length is short [54,55,56]. In addition to NGS, third-generation sequencing allows for long-read sequencing of individual RNA molecules [57]. Thus, third-generation sequencing is free from the shortcomings generated by PCR amplification and read mapping. It can greatly reduce the false positive rate of splice sites and capture the diversity of transcript isoforms [57]. Single-molecule sequencing platforms are comprised of Pacific Biosciences (PacBio) single-molecule real-time (SMRT) sequencing [58], Helicos single-molecule fluorescent sequencing [59], and Oxford Nanopore Technologies (ONT) nanopore sequencing [60]. miRNA-Seq is the last major approach in terms of accuracy and of discovery of previously uncharacterized miRNAs. However, a series of disadvantages, such as the high costs and the time-intensive process for assay design, running, and data analysis cannot be overlooked.

## 3. Nanostructured Biosensors and Nanomaterials

The advent of nanotechnology has allowed the fabrication of nanostructured biosensors which are widely exploited in the diagnostic field. Performing fast-response diagnostic and prognostic tests provides an early diagnosis at the initial stages of the disease.

In general, a biosensor is defined as a device composed of a recognition system and a transducer element that provide analytical information [13]. Currently, biochips and biosensors are gaining more attention due to their biocompatibility, ease to use, safety, and lack of sample preparation and isothermal reactions [61]. Once the target analyte has been recognized, a variety of signals can be generated by the transducer such as optical (e.g., colorimetric, fluorescent, Raman scattering), electrochemical (e.g., amperometric, voltametric, impedimetric, and potentiometric signals), chemo-luminescent, and so on [62,63].

**Figure 1:**
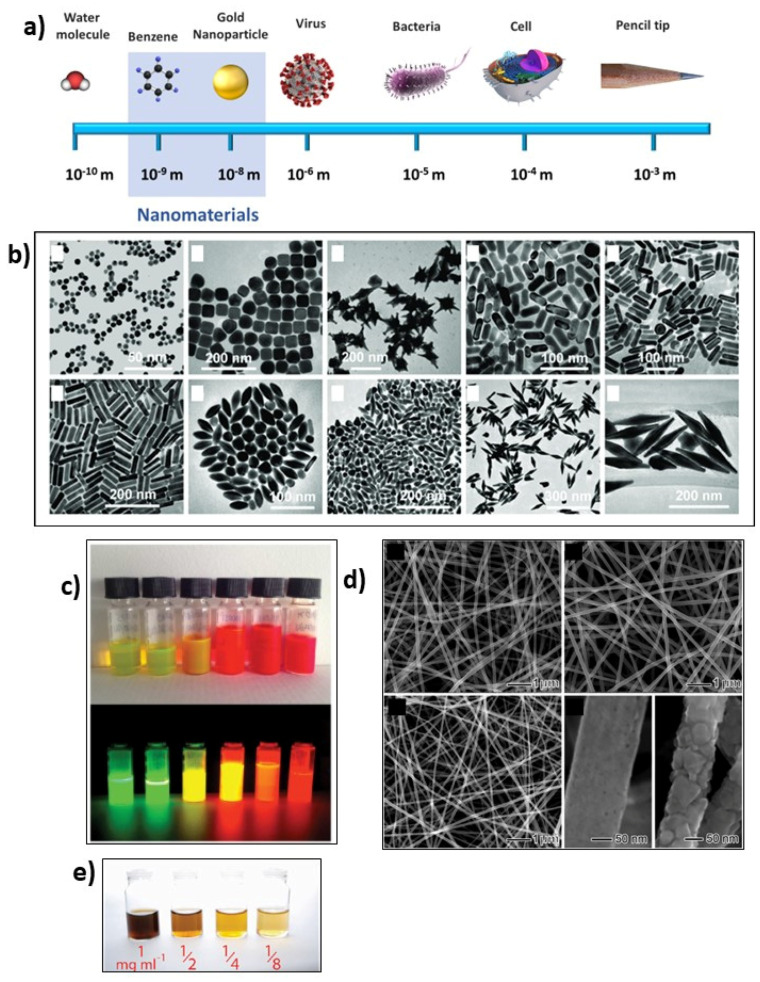
(**a**) Nanoscale illustrating the nanomaterial size compared with small and larger molecules. (**b**) Gold nanoparticles with different size and shape (on the top from left to right: nanospheres, nanocubes, nanobranches, nanorods (24 ± 0.3), and nanorods (1.5 ± 0.3); on the bottom from left to right: nanorods (4.6 ± 0.8), nanobipyramids (1.5 ± 0.3), nanobipyramids (2.7 ± 0.2), nanobipyramids (3.9 ± 0.2) and nanobipyramids (4.7 ± 0.2). Adapted with permission from Ref. [77]. Copyright (2008). American Chemical Society. (**c**) CdSe/CdS/ZnS quantum dots (QDs) before (**top**) and after UV irradiation (**bottom**), showing an emission between 530 nm and 628 nm. Adapted with permission from Ref. [78]. Copyright (2013). American Chemical Society. (**d**) Electrospun nanofiber as an example of nanostructured polymers (on the top from left to right PVP and amorphous TiO_2_ nanofibers, ceramic nanofibers of anatase; on the bottom from left to right: ceramic nanofibers of rutile and high magnification of ceramic nanofibers of anatase and of rutile). Adapted with permission from Ref. [79] Copyright (2017). American Chemical Society. (**e**) Graphene oxide at different concentrations, resulting in a color change of the aqueous solution. Adapted with permission from Ref. [80]. Copyright (2019). American Chemical Society.

To better understand the function of nanostructured biosensors, it is mandatory to introduce the concept of a “nanomaterial”. A nanomaterial is described as a small material whose size is in the 1–100 nm range (Figure 1a). Nanomaterials (NMs) are emerging since they display new and improved properties compared to their bulk counterparts. Based on their dimension they can be classified as:

- Zero-dimensional (0-D): the three dimensions are in the nanoscale range;
- One-dimensional (1-D): just one (of the three) dimension is in the nanoscale range;
- Two-dimensional (2-D): two (of the three) are in the nanoscale range;
- Three-dimensional (3-D): bulky material, not in the nanoscale range [64].

NMs can also be classified based on their chemical composition, such as metal, non-metal, metal oxides, semiconductors, but also silicates, carbonates, and nitrides [65] (Table 1).

The synthesis of NMs is accomplished mainly through top-down and bottom-up approaches. The former is based on the use of externally controlled devices that direct NMs assembly, controlling their shape and order; the latter is a technique where molecular elements, such as atoms, molecules, and clusters organize themselves in more complex structures (e.g., colloidal synthesis of nanoparticles) [66].

The large surface–volume ratio, together with the large number of functional groups and active sites on the surface of NMs, enable the immobilization of several biomolecules providing an increased absorption and catalytic activities [19,67,68]. 

The possibility to adjust NMs size is exploited to obtain desirable properties. Reducing NM size increases the surface volume that induces an increased loading capacity. Generally, most of the properties of NMs are shape- and size-dependent, such as catalytic [69], magnetic [70], electrical [71], and optical properties [72]. 

This review aims to elucidate the role of electrochemical and optical biosensors in miRNA detection. We will focus on the electrochemical and optical properties of NMs used for biochip fabrication. In detail, four classes of NMs will be discussed: metallic nanoparticles (MNPs), graphene oxide (GO), quantum dots (QDs), and nanostructured polymers. Due to their antibacterial, conductive, and optical properties, as well as their medical and therapeutic applications, noble metal nanoparticles (NPs), such as gold (Au) (Figure 1b) and silver (Ag) NPs, together with other metal NPs (based on copper (Cu), platinum (Pt), magnetite NPs (Fe_3_O_4_), etc.) play a significant role in biosensor fabrication. In general, NPs display enormous advantages such as optimized synthesis process (easy preparation, few step synthesis, small reaction volume) and easy surface functionalization [73]. 

Graphene oxide (GO) is considered another interesting NM suitable for the construction of biosensors for its unique structure which is characterized by the presence of different functional groups (epoxy, hydroxyl, and carboxyl groups) on its surface, enabling easy functionalization [74] (Figure 1e).

In addition, quantum dots (QDs) are a class of NMs that exhibit peculiar optical and conductive properties [75] (Figure 1c). Nanostructured polymers have emerged, thanks to their thermal, mechanical, and optical features, as functional tools that can be easily combined with nanoparticles and other NMs for construction of biosensors [76] (Figure 1d). 

Exploiting the chemical stability, conductivity, electrical, mechanical, and optical properties of NMs, chemical, electrochemical, and optical biosensors are fabricated.

Understanding which type of sensing strategy has to be used is crucial for the identification of particular biomarkers.

**Table 1:** Schematic representation of the features of nanomaterials.

Nanomaterials (Nms)	Classification Based on Dimension	Chemical Structures	Properties	Detection Strategy	Advantages	References
**Metallic Nanoparticles (MNPs)**	Zero-dimensional (0-D)	Noble metal such as gold (Au) and silver (Ag), but also inorganic metal (e.g., copper Cu, platinum Pt) or metal oxide (magnetite Fe_3_O_4_)	Biocompatibility, antibacterial, conductive, and optical properties	Electrochemical, optical	Optimized synthesis process, easy surface functionalization, large surface area–volume ratio	[64,73,81,82,83,84,85,86,87,88,89,90]
**Graphene Oxide (GO)**	Two-dimensional (2-D)	Several surface functional groups: epoxy, hydroxyl, and carboxyl	Thermal, mechanical, electric, and electrochemical properties	Electrochemical, optical	Easy functionalization process	[64,74,91,92]
**Quantum Dots (QDs)**	Zero-dimensional (0-D)	Semiconductor nanocrystals usually composed by II-VI group elements (Cd, Se, S, Te) and III-V group elements (In, P, As) and IV-VI group elements (Pb, Se)	Optical and conductive properties	Electrochemical, optical	Large surface area–volume ratio	[64,75,93,94]
**Nanostructured Polymers**	One-dimensional (1-D), Two-dimensional (2-D), Three-dimensional (3-D)	Mixtures of different polymers, low molecular weight chemical compounds, colloidal nanocrystals, carbon nanotubes, etc., incorporated in nanofibers or polymeric matrix	Conductivity, biocompatibility, mechanical, thermal, electronic, optical, and magnetic properties	Electrochemical, optical	Large surface area–volume ratio, fast mass-transport, easy functionalization, easy combination with nanoparticles and other NMs	[64,76,95,96,97]

## 4. MiRNA Detection via Nanostructured Biosensor: Molecular Amplification Techniques

As previously pointed out, standard detection techniques (real-time-qPCR, Northern blot, microarrays, and miRNA-Seq) are costly, time-consuming, and not able to reach a high sensitivity to detect poorly concentrated analytes [14]. Nanostructured biosensors offer many advantages as discussed above. Nowadays, several strategies are emerging as novel tools able to potentiate the efficiency of biosensors for detecting miRNAs. Depending on their approaches, they can be classified as hybridization-, separation-, or amplification-based techniques and so on. Since miRNAs are present at very low concentrations in the range of attomolar [aM] to femtomolar [fM] in body fluids, one of the major challenges for their detection accomplishing a high sensitivity and selectivity. Therefore, the development of new approaches and innovative methods are needed to reach a better low limit of detection (LOD) [98].

In this view, amplification techniques represent a solid approach able to extend miRNAs short sequence (≈18–22 nucleotides) and to increase their concentration, enhancing the specificity of the target binding and the signal transduced by the biosensor as a consequence [16]. In the following paragraphs, some of the principal amplification techniques for miRNA detection are discussed, explaining their mechanisms of action.

### 4.1. Hybridization Chain Reaction

**Figure 2:**
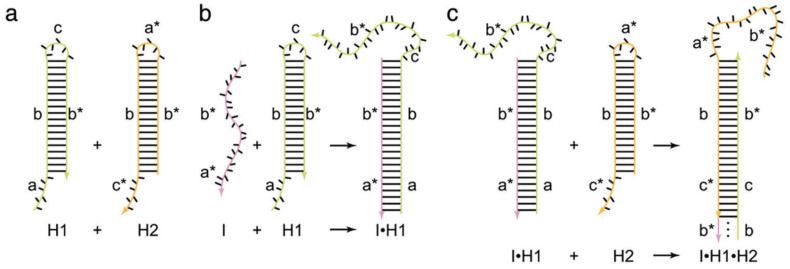
Hybridization chain reaction (HCR). Letter that are marked with * indicate that are complementary to the corresponding unmarked letter.(**a**) Stable DNA hairpins, H1 and H2. (**b**) H1 hybridization with the initiator target (I). (**c**) H1 free domain interacting with H2 and the formation of a nicked double helix. Adapted with permission from Ref. [99] Copyright (2004). The National Academy of Sciences. “Open access article under the Creative Commons Attribution License”.

Demonstrated for the first time by Drick and Pierce in 2004 [99], hybridization chain reaction (HCR) is an enzyme-free amplification technique designed to amplify short oligonucleotide sequences. HCR consists of two DNA hairpins (H1 and H2) that are kept in a kinetic trap (Figure 2a), so they are stable in the absence of the initiator (I), such as a target miRNA (Figure 2b). DNA hairpins (H1 and H2) are partially complementary to each other and have complementary regions to I (Figure 2b). When the (I) is present, it binds to an H1 complementary domain and the H1 hairpin structure unwinds, exposing a free domain that can interact with the other DNA hairpin, H2 (Figure 2b). The reaction leads to the release of the initiator and to the formation of a nicked double helix (Figure 2c) [100]. 

The effectiveness of this technique has been explored so far. Shao and colleagues [101] have designed a SERS biosensor consisting of gold nanowire vesicles deposited silver nanoparticles and HCR to detect miR-141, a well-known biomarker associated with breast and prostate cancer. A femtomolar level of detection (0.03 fM) was reached, indicating that the use of the SERS biosensor together with the HCR technique represents a winning strategy for detecting miRNAs.

#### 4.1.1. Exponential Amplification Reaction

**Figure 3:**
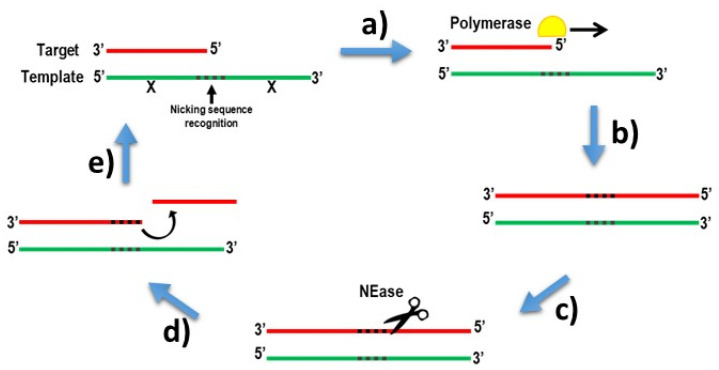
Exponential amplification reaction (EXPAR) cycle. (**a**) Polymerization of target using the template. (**b**) Extended target bearing nicking recognition sequences. (**c**) Cleavage by NEase enzyme in proximity of the target nicking recognition sequences. (**d**) Generation of the nicked target segment. (**e**) Initiation of a new EXPAR cycle by the generated target.

The exponential amplification reaction (EXPAR) was introduced by Ness’s group in 2003 [102] and represents a high sensitivity isothermal amplification strategy (Figure 3). EXPAR is formed by a DNA template (X’–X’) which is composed of two repeat sequences (X’), complementary to the target, separated by a nicking endonuclease (NEase) (-); a DNA polymerase and deoxynucleotides triphosphate (dNTPs).

EXPAR’s general mechanism is based on an amplification reaction, catalyzed by the DNA polymerase (Figure 3a) which is triggered by the presence of the target sequence (DNA, RNA, or miRNA sequence) that hybridizes to the DNA template. Once the polymerization is concluded, nicking enzyme recognition sequences are formed in the extended target (Figure 3b). The nicking endonuclease (NEase) cleaves in proximity of the recognition sequences (Figure 3c). This event generates a nick and the displacement of a segment of the extended strand (Figure 3d). The cleaved site can continue its elongation mediated by DNA polymerase (Figure 3e). The generated DNA strand can be a new target and starts an amplification cycle of another DNA template [103].

EXPAR products can be detected through fluorescence probes [104] or colorimetric assay [105] that generate a simple color change of the solution, through chemo-luminescence detection using specific enzymes [106], or using the hybridization to reporter probes that generate a surface-enhanced Raman signal (SERS) [107]. Recently, EXPAR has shown to be successful in the detection of exosomal miRNAs. In particular, Qian et al. [108] proposed a point-of-care (POC) device for the easy detection of miRNAs. The strategy consisted of an on-chip amplification reaction for the multiplexed quantification of exosomal miRNAs. The chip is composed by a flow cell dedicated to the extraction of the vesicles and their lysis, and another flow cell where the quantification of the analyte is performed via EXPAR. Once the exosomes are extracted from cell cultures using magnetic microbeads, they lysate and the resulting miRNAs are transferred on the chip. Here, the measurement of target miRNAs is carried out via the amplification steps and the fluorescence signals are analyzed through an IoT reader, with the results being proportional to the concentrations.

#### 4.1.2. Rolling Cycle Amplification

**Figure 4:**
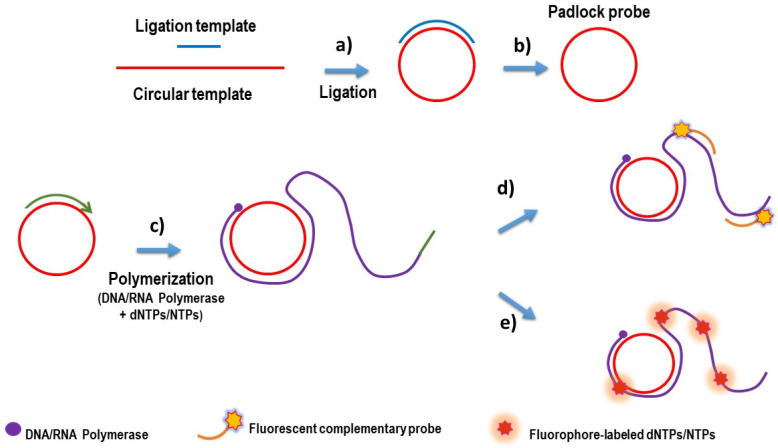
(**a**) Ligation of target template and (**b**) formation of the Padlock probe. (**c**) Amplification of the target sequence catalyzed by DNA/RNA polymerase, with the incorporation of dNTPs/NTPs, generating the concatemer with tandem repeats. Detection via (**d**) fluorescent probes or via (**e**) fluorophore-labelled dNTPs/NTPs.

Rolling cycle amplification (RCA) was first used by Jonstrup in 2006 [109] and it represents another valid strategy for the detection of miRNAs. RCA is an isothermal process that offers the possibility to amplify a target sequence (DNA and/or RNA) (Figure 4). Target DNA or RNA act as primers that generate, through an enzymatic ligation, a circular probe, called a Padlock probe (Figure 4a,b). Once hybridization of the target and the padlock probe has occurred, a polymerase enzyme (Phi29 DNA polymerase or T7 RNA polymerase), in the presence of deoxynucleotide triphosphates (dNTPs) or nucleotide triphosphates (NTP), starts the amplification leading to a concatemer consisting of tandem repeats (Figure 4c). To analyze the product, complementary fluorescent probes are used (Figure 4d) or fluorophore-labelled dNTPs/NTPs (Figure 4e) are incorporated during the amplification allowing a fluorescent-based technique visualization [110,111,112].

Due to its versatility and specificity, RCA has been combined with other techniques such as the clustered regularly interspaced short palindromic repeats (CRISPR). More specifically, RCA and CRISPR/Cas12 have shown to be a great machinery for miRNA detection [12]. Once the amplification by RCA is completed, the released product, complementary to the padlock probe, can be processed by the Cas12. After the recognition of tandem repeat (P’), the CRISPR/Cas12 system activates and induces a “trans cleavage” near the labelled DNA probe (reporter), whose fluorophore is no longer quenched, resulting in the emission of a fluorescence signal (FRET).

#### 4.1.3. Duplex-Specific Nuclease

**Figure 5:**
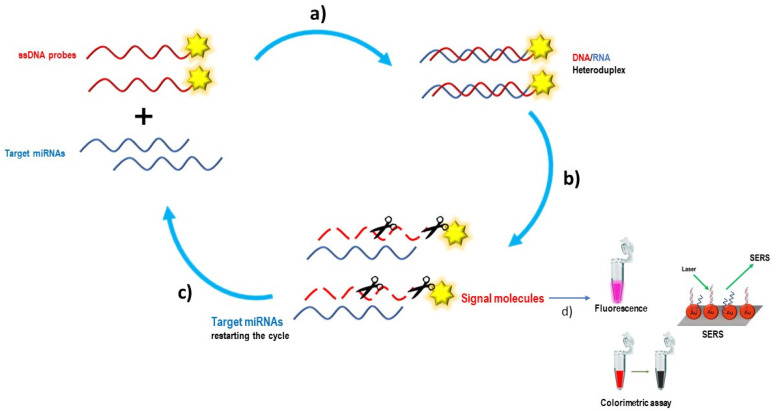
Duplex-specific nuclease amplification cycles. (**a**) Single-strand DNA (ssDNA) hybridized with target miRNAs, and formation of the DNA/RNA heteroduplex. (**b**) Endonuclease cleavage of DNA/RNA heteroduplex. (**c**) Released miRNAs restarting the cycle. (**d**) Generated signal molecules detected via different kinds of biosensors.

The duplex-specific nuclease (DSN) assay was designed as a method for miRNA detection in 2002 by Shaghin and colleagues [113]. It implies the use of an endonuclease extracted from hepatopancreas Red King (Kamchatka) crab. DSN shows a certain specificity in the cleavage of double-stranded DNA and DNA/RNA heteroduplex (Figure 5b). The DSN-mediated degradation of a DNA/miRNA heteroduplex produces a free miRNA that can bind the DNA probe and restart the amplification/breakage cycle (Figure 5b,c), enhancing the signal. These latter free miRNAs enable the release of “signal molecules” that are proportional or inversely proportional to the target miRNA. Based on the type of “signal molecule”, it is possible to classify different DSN-based biosensors in fluorescent, colorimetric, surface-enhanced Raman scattering (SERS), and so on (Figure 5d) [114].

A signal amplification strategy was proposed by Zhan’s group for the detection of the overmentioned miR141, a known cancer biomarker [115]. When the target miRNA is present, it forms a duplex with a molecular beacon (MB1) which undergoes conformational change, becoming available for DSN cleavage. Once the DSN recognizes the duplex, the latter is cleaved by its nuclease activity. Then, the target miRNA and DNA are released. The generated DNA fragments can bind to the other MB2, forming a G-oligomer that acts as a fluorescent transducer together with malachite green (MG). The fluorescence signal is proportional to the quantity of the detected target and its intensity is enhanced more than 200% using the DSN amplified method.

## 5. Probes Immobilization and Functionalization Strategies

Surface biosensor functionalization promotes the enhancement of selectivity and sensitivity for the target analyte. The combination of nanostructured material functionalized with oligonucleotide probes and amplification assay represent a suitable strategy to detect miRNAs. Their large surface area–volume ratio enables the immobilization of a number of “capture probes” complementary to the target, increasing the chances of interaction.

**Figure 6:**
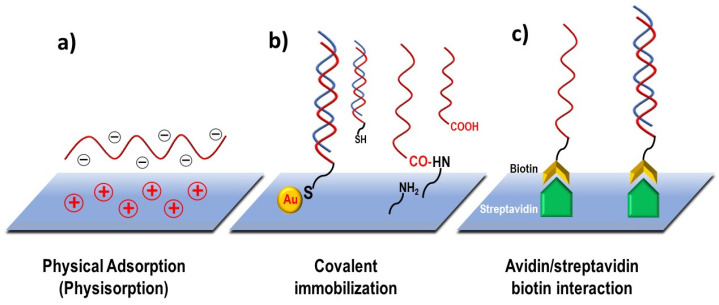
Oligonucleotide probe immobilization on a surface. (**a**) Physical adsorption occurring through electrostatic interaction. (**b**) In covalent attachment, the probe is immobilized via chemical bonding. Chemisorption (on the **left**) occurs between gold (Au) and a thiol-modified probe. On the right, a carboxyl-modified probe interacts with the amino group of the support. (**c**) In Avidin/streptavidin–biotin, the noncovalent bonding between streptavidin–biotin is preferred over avidin. Biotin is usually linked at one end of the probe, while streptavidin is attached on the support, as shown in the figure.

Probe immobilization consists of coating the surface of the nanostructured device. The main immobilization strategies comprise of physical adsorption, covalent binding, and avidin/streptavidin biotin interaction (Figure 6a–c).

Physical adsorption, or physisorption (Figure 6a), is a simple immobilization approach since it does not require any modification of the probe and is based on electrostatic affinity. In the case of DNA probes, taking advantage of negative charges, an attractive interaction with the positive charges on the surface of a support is generated [116]. An example is provided by the work of Gong and colleagues. They designed an electrochemical biosensor for HIV-1 gene detection using a single-stranded DNA (ssDNA) probe attached on a polyaniline/graphene (PAN/GN) glassy carbon electrode (GCE) through a π–π stacking interaction. When the target gene is present, it hybridizes with the ssDNA capture probe, resulting in a double-stranded DNA (dsDNA). Selectivity, sensitivity, and specificity towards the HIV-1 gene was demonstrated, reaching an LOD value of 1.0 × 10^−16^ M [117].

One of the major limits of physisorption is that its principle is based on a weak interaction. As a result, physical absorption is affected by changes in the pH, temperature, and ionic strength. Conversely, covalent bonding shows a superior degree of stability in terms of attachment to the surface and affinity with the target molecule (Figure 6b). Chemisorption and covalent attachment are two types of immobilization that are mainly performed for their simplicity (Figure 6b). Chemisorption is a phenomenon that involves the interaction of the thiol-modified probes with gold (Au) surface. The resulting gold (Au)–sulfur (S) covalent bonding represents a strong covalently immobilization that enables a great stability of the attached probe [118]. Due to the Au–S strong interaction and their large surface area–volume ratio, Au nanoparticles allow an easy probe immobilization. Rotz et al. describe this advantage in their work in which they propose a new strategy for labelling spherical nucleic acid (SNA) conjugates by synthetizing Gd(III) labelled DNA Au nanoparticles (AuNPs). These SNAs consist of Au nanoparticles functionalized via Au–S bonding with 3′-thiol-DNA probes loaded onto AuNPs surface. On SNAs, the probes are oriented to cover the AuNPs surface area. In this specific work, they proceed with a co-functionalization using Gd(III), favored by AuNPs with a high surface density [119]. Covalent attachment includes the chemical modification of the nanostructured support. Immobilization of probes on a solid support can be promoted by covalent interaction of several functional groups present on the surface. Some examples are aldehyde [120], epoxy [121], isothiocyanate, and sulfonic [122] groups. For instance, chitosan nanocomposites have emerged as a potential matrix allowing probe immobilization. Using its amino and carboxyl group, strong and stable covalent bonds are generated with probes or several nanomaterials (metal nanoparticles, graphene nanosheet, quantum dots, carbon nanotubes, etc.) [123].

One of the covalent attachment chemistries mostly used is the carbodimmide binding, consisting of 1-ethyl-3-(3-dimethylaminopropyl) carbodiimide (EDC) as a coupling agent and N-hydroxysuccinimide (NHS). EDC activates the carboxyl groups which are then able to bind the amine groups, forming a covalent amide bond. EDC/NHS is usually combined with other immobilization strategies, such as the streptavidin–biotin interaction which consists of a noncovalent approach. Indeed, in addition to covalent attachment, the streptavidin–biotin interaction is a largely used method for probe immobilization (Figure 6c). Biotin, a small molecule, binds to streptavidin (SA) (K_d_ ≈ 10^−14^ M) resulting in a strong noncovalent binding. SA is a tetrameric protein, exposing four binding sites available for biotin interaction. SA binding is favored over avidin since this latter has a lower binding affinity when biotin is linked to another molecule [124]. The combination of the two techniques has been described by Liu and co-workers to perform a new functionalization for SERS sensing. Graphene-encapsulated gold (Au) nanoparticles (GNPs) are attached to single-walled carbon nanotubes (SWCNTs) using Streptavidin–biotin. Firstly, GNPs are treated with oxygen plasma to obtain carboxyl functional groups on the surface. Then, through EDC/NHS carbodimide chemistry is adopted to form amine reactive groups which further react with an amino terminal biotin. Eventually, this latter can bind to streptavidin modified SWCNTs resulting in streptavidin–biotin noncovalent bonding [125]. SA–biotin has been exploited to anchor DNA probes on a solid surface for miRNA detection, as mentioned in a recent published work [126]. These biochips are based on RNA/DNA hybridization via the assembly of five DNA probes modified with fluorophores. One of these is 5′-biotin labelled to ensure its anchoring on a streptavidin-modified microscope slide.

## 6. Electrochemical Biosensors for miRNA Detection

**Scheme 1:**
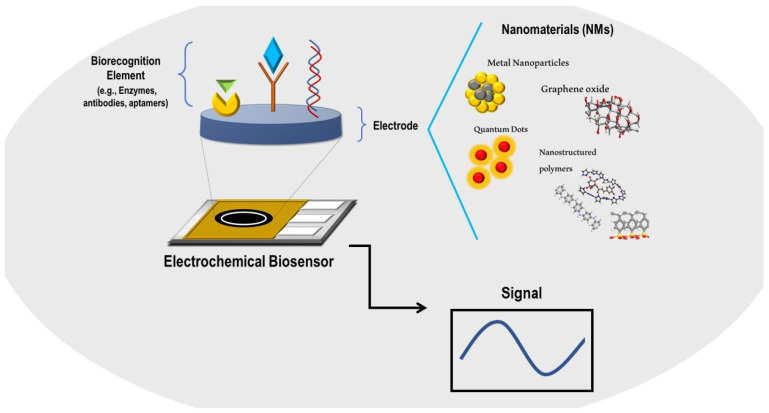
Schematic representation of an electrochemical biosensor.

Due to their simplicity, low-cost, biocompatibility, easy use, and rapid response (signal read out), electrochemical biosensor devices have gained attention in prognostic and diagnostic fields in the past few years, consolidating their role as a sensible approach for miRNA detection (Scheme 1). 

The first electrochemical biosensor was introduced by Clark and Lyons in 1962, in an attempt to monitor glucose blood concentrations. They created the “oxygen electrode” modified with glucose oxygenase immobilized on its surface. When oxygen was present, the enzyme was able to oxidize glucose into peroxide. The decrease of oxygen concentration was proportional to the amount of detected glucose [127]. Generally, electrochemical biosensors are composed of a biological sensing element (a protein, an enzyme, a nucleic acid, an antibody, etc.), that upon interaction with the target analyte produces a signal that is converted by a transducer element into an electric signal. Eventually, the signal is processed and “translated” by computational software into a parameter (Figure 6) [128]. Amperometric, voltametric, impedimetric, and potentiometric techniques represent the various transduction approaches used to measure the signal in electrochemical biosensors [129]. Enzymes represent the most used biological sensing elements for their catalytic properties, having a high affinity for a specific substrate, or for their role as labels for different molecules (antibodies, antigens, oligonucleotides, and so on) [130]. 

Electrochemical biosensors generally include three elements: a reference, an auxiliary, and a working electrode. The reference electrode (Ag/AgCl) has to maintain a stable potential, so it cannot be in proximity of the site of interaction between the analyte and the biorecognition element. Conversely, the counter or auxiliary electrode is in contact with the electrolytic solution, and the current that passes through the solution is measured by the change of potential of the working electrode that is closer to the reaction and acts as a transducer [130]. The electrode is the key component to ensure the functioning of the electrochemical biosensor. Over the past decade, several electrode modifications were introduced. For instance, the fabrication of screen-printed electrodes (SPE) is a well-grounded technique of printing high viscous inks on a plastic or ceramic substrate [131] and it is employed as a way to meet the specificity, sensitivity, and reproducibility requirements in electrochemical biosensor, while maintaining a low cost of production [17]. 

Moreover, with the advance of nanotechnology, the miniaturization at the nanoscale level and low-cost large-scale production have contributed to making electrochemical biosensors solid alternative methods for miRNA detection. They possess a large area–volume ratio, loading capacity, and allow easy surface modification, most of the time using DNA capture probe generating DNA/RNA complexes. To improve properties such as magnetism, electrical conductivity, and reactivity, NMs are implemented to modify electrodes or as electrodes themselves, increasing immobilization efficiency and ensuring a high level of sensitivity since they are able to detect the low concentrations of target analytes [17,18] (Scheme 1). Here, we reported four classes of functional nanomaterials that are employed for the modification and nano-miniaturization of electrodes in electrochemical biosensors designed for miRNA detection (Table 2).

### 6.1. Metallic Nanoparticles

Metallic NPs have been employed as solid substrates for electrochemical biosensors, boosting both transfer efficiency and surface area–volume ratio [81]. They can be electrodeposited onto an electrode surface or can act as electrodes themselves.

A general description of some widely used metallic NPs and their most recent applications for miRNA detection (Table 2) is provided in the following paragraph.

#### 6.1.1. Gold Nanoparticles

Due to their remarkable biocompatibility and easy electron transfer between immobilized molecules and electrode surface, gold NPs (AuNPs) are the most extensively employed metal nanoparticles in electrochemical biosensors. When the sensing electrode is modified with AuNPs, the electrical signal should be strong in order to achieve a good signal–background ratio [82] (Table 1). In addition, the large surface capacity makes AuNPs appropriate substrates for probe immobilization aimed to sense the analyte of interest. In the case of miRNA identification, AuNPs are functionalized with oligonucleotide probes whose sequences are complementary to the target, resulting in their hybridization [141]. Combined with the possibility to modify electrodes (e.g., electrodepositing AuNPs on SPEs surface (Au-SPE) [142]) and exploiting AuNPs molecular recognition properties, high performance electrochemical biosensors for miRNA detection can be obtained. 

The modification of electrodes using AuNPs was reported in Yammouri et al. [132]. In this specific study, a great amount of thiolated capture probes, labeled with methylene blue (MB), were immobilized on AuNPs electrochemically loaded on the surface of a pencil carbon electrode (PGE), which was additionally modified with carbon black (CB). The present measurement device PGE/CB/AuNPs was implemented for the detection of miR-21. When the target miR-21 hybridized to the capture probe, the decrease of oxidation of MB was exploited to demonstrate that the specific and selective interaction had occurred. They justified the decrease of MB oxidation as a consequence of the conformational change upon the target/probe interaction. The electrochemical biosensor reached an LOD of 1 fM. 

**Figure 7:**
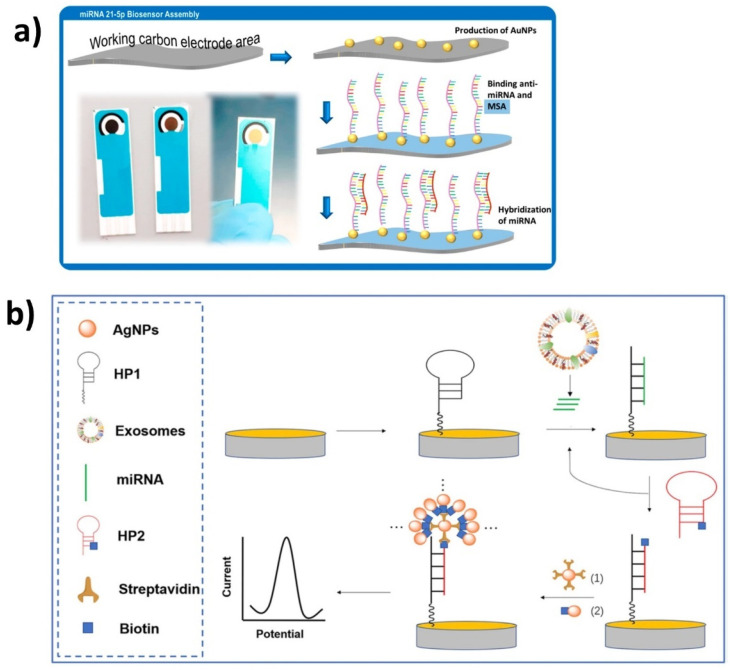
(**a**) Sensing of miR-21 using a printed carbon electrode functionalized with AuNPs. Adapted with permission from Ref. [83]. Copyright (2022). MDPI-open access article under the Creative Commons Attribution License. (**b**) Schematic representation of electrochemical biosensor based on a gold-modified electrode. Biotinylated AgNPs, deposited after interaction with target miR-21, interacting with streptavidin-modified HP probes. Adapted with permission from Ref. [84]. Copyright (2020). Elsevier.

Achieving lower LODs is the main challenge of designing biosensors for miRNA detection due to their low concentration. In a recent work published in 2022 [83], an attomolar (aM) detection limit was obtained for miR-21-5p. The strategy proposed comprised of the use of a screen-printed carbon electrode (C-SPE) modified with AuNPs synthetized in situ (Figure 7a). On the latter, thiolated anti-miRNA complementary strands were immobilized via chemisorption. AuNPs electrodeposition time was optimized to obtain a modified electrode suitable for probe functionalization and to increase the electrical and conductive properties of the device. After hybridization with the target, a great change in electrochemical impedance spectroscopy (EIS) was recorded, with respect to cyclic voltammetry (CV) and square wave voltammetry (SWV). During calibration of miR-21-5p in buffered solution, this low-cost, selective, and sensitive system reached 4.31 aM and provided a linear response in human blood serum as well.

#### 6.1.2. Silver Nanoparticles

A higher capacity, if compared to AuNPs, in electrochemical biosensors has been demonstrated by silver NPs (AgNPs). AgNPs’ properties make them suitable nanomaterials in electrochemical sensing. They are easy to prepare and modify, have a large area–volume ratio, substantial electrochemical reactivity, sharp oxidation peaks, and a low reduction potential, unlike AuNPs [82,143] (Table 1). A femtomolar (fM) concentration of the above-mentioned miR-21 was detected via an electrochemical biosensor based on the use of a gold electrode, where streptavidin-modified AgNPs were deposited (Figure 7b) [84]. More specifically, the mechanism of function consisted of a cyclic amplification of the target miRNA which hybridized to a hairpin loop (HP1) immobilized on the gold electrode via an Au–S bond. Another HP2, biotin-modified, was added, resulting in the displacement of the miRNA, being able to start another amplification cycle. Streptavidin-modified AgNPs interact with biotin on HP2, generating a peak current proportional to the miR-21 concentration (LOD 0.4 fM). A similar but more complex amplification approach exploiting AgNPs’ properties was illustrated in a very recent work [134]. A Methylene blue (MB) DNA hairpin (MB-HP1) and an AgNPs-DNA hairpin (Ag-HP2) were employed as reference and signal probes, respectively. The MB-HP1 was attached to the electrode and, after the production of strands (S1) resulting from DNA walker amplification in the presence of target (miR-155), S1 bound to MB-HP1, opening the loop. The produced capture probe interacts with Ag-HP2 and the amount of AgNPs was directly proportional to the target concentration. The detection limit calculated was 3.2 fM.

#### 6.1.3. Other Metallic Nanoparticles

Along with noble metals, other metallic nanoparticles are employed for electrochemical sensing.

Copper (Cu) is a transition semiconductor metal with prestigious properties, making it one of the most used materials. Cu is characterized by easy malleability, high thermal and electrical conductivity, and great corrosion resistance [85] (Table 1). For their conductivity, CuNPs represents a valid substrate able to increase the electric signal.

For miR-222 detection, Wang et al. presented an electrochemical biosensor based on an HCR amplification strategy generating DNA concatemers as template for CuNPs synthesis to be used as capture probes. The electrode was modified with a reduced graphene oxide (rGO) electrode and AuNPs. When the target miR-222 was present, it interacted with the DNA templated CuNPs; a great oxidation peak was registered due to CuNPs. The overall sensing apparatus reached an LOD of 0.03 fM [136]. 

Platinum NPs (PNPs) are widely used in electrochemical reactions since they have the ability to break down hydrogen peroxide, which is produced in oxidative reactions. The electrons that are formed or absorbed in the oxidative or reductive reaction are used to generate an amperometric signal. Nevertheless, PNPs are employed not only for these kinds of reactions. A synergic effect of PNPs combined with AuNPs has been demonstrated, resulting in bimetallic nanoparticles (AuPBNPs), in the detection of miR-21 [86]. AuPBNPs were used to immobilize the DNA capture probe via glutaraldehyde linker on a TFO, previously hydroxylased using APTS. The interaction with target miR-21 was electrochemically measured via a DPV assay. The biosensor showed an LOD of 1 fM.

### 6.2. Graphene Oxide

**Figure 8:**
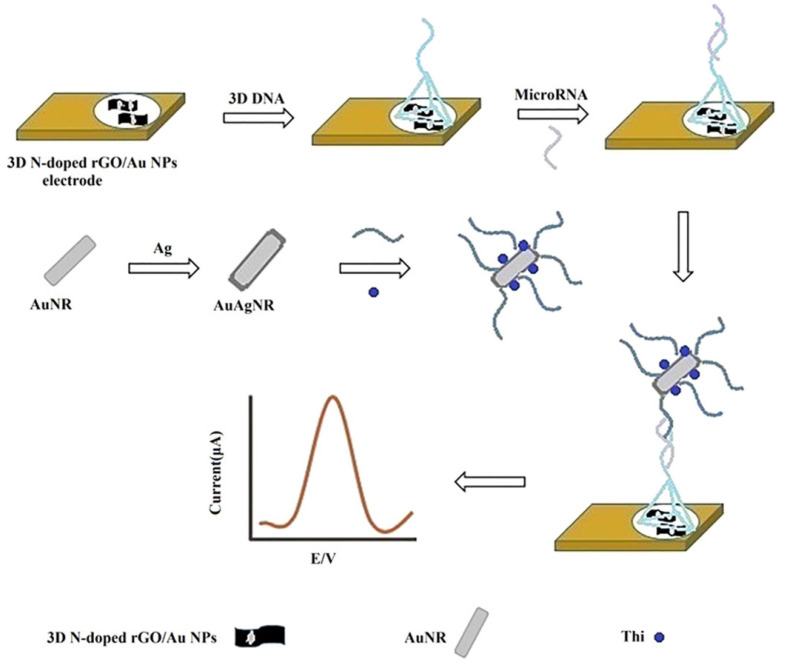
Schematic representation of the assembling and application of an electrochemical biosensor based on (N)-doped rGO/AuNPs-modified electrode for miR-155 detection. Adapted with permission from Ref. [135]. Copyright (2018). Elsevier.

Different from graphene, which possesses a planar hexagonal structure, graphene oxide (GO) represents a particular two-dimensional (2-D) carbon-based nanomaterial. The presence of oxygen makes GO easy to be functionalized. It contains several epoxy, hydroxyl, and carboxyl groups. GO shows excellent thermal, mechanical, electrical, and electrochemical properties [91] (Table 1). Due to its characteristics, GO represents a promising nanostructure for electrochemical biosensor fabrication. It is possible to modify the working electrode using nanosheets of reduced GO (rGO), as it was demonstrated by Torul et al. [139]. The paper-based electrochemical biosensor for detecting miR-155 and miR-21 was constructed using a working electrode modified with rGO covered with AuNPs (AuNPs/rGO). Complementary thiolated DNA probes to target miRNAs were immobilized onto AuNPs. Differential pulse voltammetry (DPV) was measured in the absence and presence of the target, and the difference of the signal indicated whether the hybridization occurred or not. In the same work, they also used a paper electrode-modified molybdenum disulfide (MoS_2_) AuNPs/MoS_2_, but the sensitivity they reached was higher using AuNPs/RGO. However, in contrast with lower LODs previously mentioned in this review, they obtained LODs for both miRNAs that were at the nanomolar level (nM) (Table 1). A lower limit of detection was calculated in another work (1 pM) [135], where the design of the biosensor was based on surface electrode modification with 3D nitrogen (N)-doped reduced graphene oxide/AuNPs (Figure 8). Doping is a method to modify surface nanomaterials without establishing great alteration. MiR-155 was detected through tetrahedral DNA probes, whose thionine reduction provided a current response upon the binding with the target. 

### 6.3. Quantum Dots

**Figure 9:**
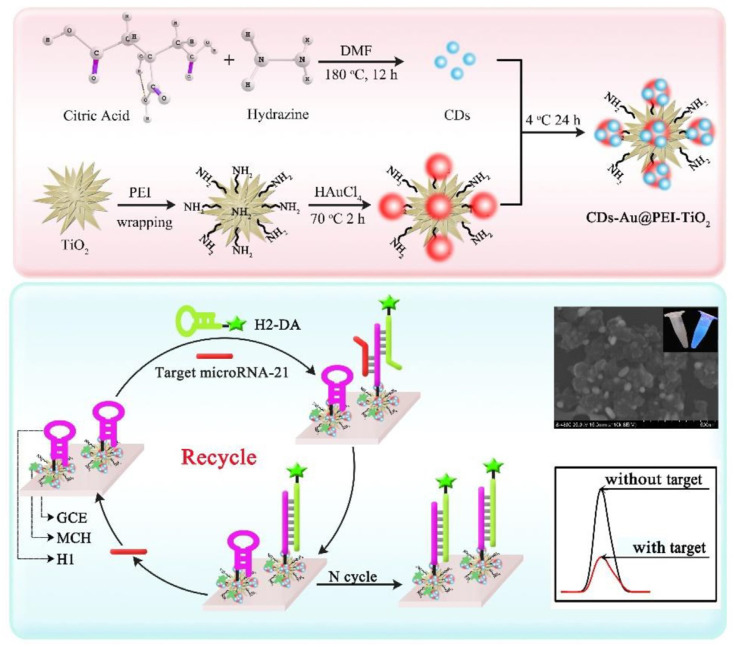
(**Upper panel**) Synthesis of CQDs-Au-PEI@TiO_2_. (**Lower panel**) Catalytic hairpin (CHA) amplification cycles generated after hybridization of the target with a hairpin strand immobilized on a self-catalyzed CQDs-Au-PEI@TiO_2_ anode. Quenching groups of the amplified sequence determines the decrease in the ECL signal. Adapted with permission from Ref. [133]. Copyright (2019). Elsevier.

Quantum dots (QDS) are a particular class of NMs. They are used to improve electron transfer, acting as electrode modifiers. They have also been used to increase the loadings of immobilized bioreceptors on the electrode surface due to their large surface–volume ratio and several functional groups [93] (Table 1). Carbon quantum dots (CQDs) were used by Zhong’s group for the realization of an electroluminescence (ECL) biosensor for quantification of miR-21. Exploiting their electric and chemiluminescent properties, CQDs were functionalized on the electrode, generating a powerful ECL luminophore. Hairpin strands were immobilized on the self-catalytic CQDs-Au-PEI@TiO2 anode and, when bound to the target, they started catalytic hairpin (CHA) amplification cycles to produce multiple copies of the target miRNA (Figure 9). Due to the presence of quenching groups of the generated amplified sequences, that could be captured on the electrode surface. The ECL signal decreased as miR-21 concentration increased, measuring an LOD of 0.03 fM [133].

Equally important, due to the presence of several functional groups on the surface and electrical conductivity, graphene quantum dots (GQDs) represent a suitable substrate for electrochemical biosensors. Pothipor et al. [140] described a system based on three electrodes modified with AuNPs, graphene quantum dots (GQDs), and graphene oxide (GO) (AuNPs/GQDs/GO) aimed to detect breast cancer miRNA biomarkers. In total, three different complementary probes were assembled on each electrode and upon hybridization with the target molecules, electrochemical signals were generated by three redox dyes, which were used as indicators. They were able to perform multiplex detection for miR-21, miR-155, and miR-210, reaching a femtomolar level of detection in human serum (Table 1).

### 6.4. Nanostructured Polymers

**Figure 10:**
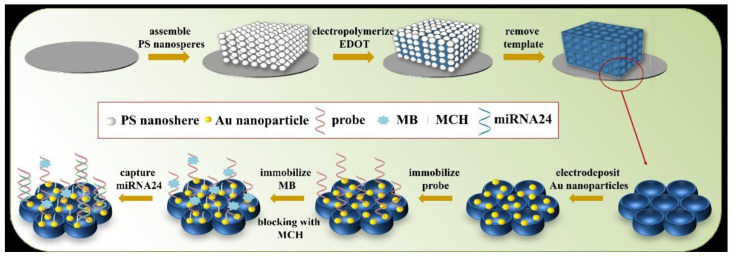
Assembling of the electrochemical biosensor proposed by Ma and colleagues [137] based on a PEDOT film where electrodeposited AuNPs are used to immobilize an MB-modified probe for capturing miR-24. Adapted with permission from Ref. [137]. Copyright (2020). Elsevier.

A new trend in electrochemical biosensors is the use of nanostructured polymers for electrode modification. In addition to the typical features of nanostructured material (large area–volume ratio, fast mass-transport, easy functionalization, and so on), nanostructured polymers display great conductivity, but also biocompatibility, as well as mechanical, electronic, and magnetic properties, making them suitable tools for electrochemical sensing. However, non-conductive polymers decorated with other NMs, such as AuNPs, graphene, and carbon-based materials, are used as well to obtain polymer-modified electrodes. Therefore, combining conductive, non-conductive polymers, and their functionalization with nanostructured material is a valuable method to improve selectivity and sensitivity [95,96] (Table 1). For instance, Ma and colleagues constructed an electrochemical biosensor for miR-24 using polymer-modified electrodes functionalized with AuNPs, able to achieve an LOD of 3.8 fM (Figure 10). Poly(3,4-ethylenedioxythiophene) (PEDOT) was used as a 3D porous film where AuNPs were electrodeposited. Taking advantage of the strong chemical bond between Au and SH- groups, thiolated DNA complementary sequences were immobilized on AuNPs, serving as capture probes for miR-24. They used Methylene blue as a redox indicator, that in the presence of the target, was displaced from the DNA probe, producing a suppression of the signal, monitored by differential pulse voltammetry (DPV) [137]. Interestingly, miR-24 detection was used as a model study in another work where conductive hydrogel was proposed as an electrochemical sensing system whose LOD was estimated to be 0.34 fM [138]. For the hydrogel preparation, polyaniline (PANI) polymers were assembled with phytic acid (PA) resulting in a PANI/PA conductive and multi-pore structure. Using the presence of amino groups of a PANI/PA-modified electrode, carboxyl-terminal DNA probes were immobilized onto the surface. A reduction in current was registered upon DNA/RNA binding.

## 7. Optical Biosensors for miRNA Detection

**Scheme 2:**
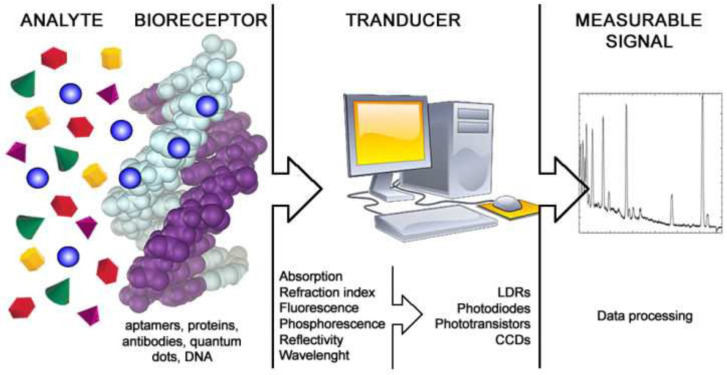
Schematic representation of optical biosensor. Adapted with permission from Ref. [144]. Copyright (2016). IntechOpen open access article under the Creative Commons Attribution License.

Optical biosensors are analytical devices that induce a fast response and easy detection of specific biological and chemical species. Upon interaction of the target molecule with the biorecognition element of the biosensor (e.g., enzyme, antibody, nucleic acid, cell, and so on), the transducer generates an optical signal which provides analytical information about the concentration of the investigated substance (Scheme 2). 

Basically, the sensing strategy can be classified as label-free and label-based. In the label-free method, a signal is generated when the analyte directly interacts at the interface of the biorecognition element. On the other hand, the label-based approach implicates the use of a label (e.g., a fluorophore, a bioluminescent molecule, etc.). Upon interaction with the target, the optical signal is colorimetric, luminescent, or fluorescent [20,145]. Different types of optical biosensors are available showing different applications. Surface plasmon resonance (SPR)-based biosensors enable reaction kinetic, equilibrium, and concentration analysis, providing qualitative and quantitative approaches. When the incident light (at a certain angle) interacts with the delocalized electrons (plasmons) of a metal surface, a reduction of intensity of the reflected light occurs, thus generating the surface plasmon resonance (SPR) effect. The binding of the biomolecule with the capture element is reported on an SPR sensogram as a change in reflectivity, angle, or wavelengths as a function of time [146]. In the case of smaller metal surfaces, such as metal NPs (MNPs), the electromagnetic radiation of the incident light induces a localized electron oscillation and the light absorbance is confined in the ultraviolet–visible (UV-vis) band. This latter event is known as localized surface plasmon resonance (LSPR). In an LSPR biosensor, a wavelength shift is recorded when the molecules of interest are captured by the recognition probe, previously immobilized on the MNPs surface [147]. Nanostructures are also employed in surface-enhanced Raman scattering (SERS) biosensors due to their ability to increment the localized electromagnetic field, enhancing the Raman signals (molecular vibrations) of the target molecules [148]. Ellipsometric biosensors allow monitoring the changing polarization of light that has been reflected [149]. Moreover, improvements in genetic recombination techniques have led to the generation of bioluminescent molecules that are usually immobilized in optical fiber that emit an optical signal in response to the binding of the analyte of interest [150]. In recent years, optical biosensors have established a significant role in miRNA quantification, since their ability to reach a low detection limit and high sensitivity and specificity were confirmed. Using optical properties of nanostructured materials, optical biosensors represent suitable devices for detecting small molecules such as miRNAs, becoming a valid alternative to old molecular strategies (Table 3) [21]. In the following paragraphs, the use of metal NPs, graphene oxide, quantum dots, and nanostructured polymers in optical sensors will be discussed.

### 7.1. Metallic Nanoparticles

As mentioned above, metal NPs, especially noble metals such as gold (Au) and silver (Ag), exhibit optical properties due to the localized surface plasmon resonance (LSPR) phenomenon. SPRs are oscillations of electrons (plasmons) in close proximity with a metal-dielectric interface. The resonances of noble metals can directly interact with light. Plasmon resonance propagation is localized on their reduced surface and MNPs act as cavity resonators. Since the optical properties are size- and shape-dependent, several synthesis strategies aim to obtain MNPs that exhibit specific optical features [87] (Table 1). Therefore, due to their ability to interact with light and their large surface area–volume ratio that enables probe immobilization, MNPs are indicated as suitable substrates for miRNA sensing (Table 3).

#### 7.1.1. Gold Nanoparticles

**Figure 11:**
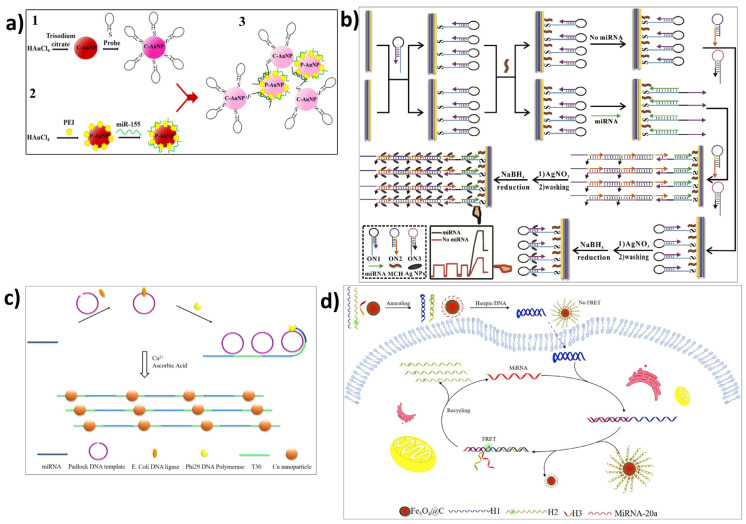
(**a**) Colorimetric detection of miR-155 using trisodium citrate-AuNPs (C-AuNPs) functionalized with thiolated hairpin probe, and polyethylenimine (PEI) capped AuNPs (P-AuNPS) used as a capture probe. miRNA recognition led to aggregation of both C-AuNPs and P-AuNPs, resulting in a color change of the solution (red–pink, absorbance at ≈530 nm). Adapted with permission from Ref. [151]. Copyright (2018). The Author(s). Published by Scientific Report. Open access article under the Creative Commons Attribution License. (**b**) Scheme of SPR-sensor for miRNA let7a detection exploiting the HCR strategy and AgNPs in situ synthesis to enhance SPR signal. Adapted with permission from Ref. [88]. Copyright (2019). Elsevier. (**c**) let7a detection using RCA and ssDNA-intercalating fluorescent CuNPs. Adapted with permission from Ref. [89]. Copyright (2020). MDPI Open access article under the Creative Commons Attribution License. (**d**) Mechanism of function of CHA-Fe_3_O_4_@C strategy: Fe_3_O_4_@C complex associated acting as quencher of H3 and H2 hairpin in absence of miR-20a. When the target was present, H3 and H2 displace and a strong fluorescence signal proportional to the miRNA concentration is generated. Adapted with permission from Ref. [90]. Copyright (2020). Elsevier.

AuNPs role as a sensing platform is due to their remarkable optical properties. For instance, being a noble metal, when AuNPs are close to each other, their surface electron oscillation (plasmons) induces a “plasmon coupling effect”, which leads to an increase of the surrounding electromagnetic field, resulting in the so-called “hotspots”. When an analyte is present at a hotspot, it is subjected to the interaction of the opposite charges of the NPs. This potentiates the weak molecular properties (Raman signals) of the absorbed molecules, resulting in surface-enhanced Raman scattering (SERS) effect, widely exploited in optical sensing [159,160]. Paramagnetic AuNPs were used as substrates in a SERS biosensor. Paramagnetic nanoparticles were coated with gold (Au@MNPs) and used for the immobilization of the oligonucleotide probe, complementary to the target miR-141. The target sequence was recognized not only by Au@MNPs, but also by AuNPs functionalized with an oligo probe carrying a Raman reporter. The resulting complex was able to improve the LOD to 100 fM [153]. Aggregation of AuNPs is another effect which leads to a change in optical properties (e.g., red shift of plasmon resonance) and is used as a sensing strategy. Hakimian et al. proposed a colorimetric detection approach for miR-155, using two groups of AuNPs [151]. The first AuNPs group (C-AuNPs), capped with trisodium citrate, was functionalized with thiolated hairpin probe; while the other AuNPs group (P-AuNPS), capped with branched polyethylenimine (PEI), was used for trapping the target miRNA (Figure 11a). The sensing strategy was based on a colorimetric assay; when the C-AuNPs and P-AuNPs groups were mixed, the probe recognition of the target caused cross-linking aggregates resulting in a color change of the solution (from red–pink to pink) and a decreased absorbance (≈530 nm). This colorimetric optical sensor achieved an LOD of 100 aM.

#### 7.1.2. Silver Nanoparticles

Several synthesis strategies enable the fabrication of AgNPs with various sizes and resulted in a plasmonic peak in the range 393–738 cm^−1^ [161]. The radioactive and non-radioactive decay generated by the movement of the electrons determines the conversion of the photon energy into thermal energy. This feature makes AgNPs suitable for diagnostic imaging applications. In addition, AgNPs have antimicrobial and antifungal properties. AgNPs also have LSPR optical properties since their conductive electrons are able to interact with the light. A SPR sensor targeting let-7a miRNA was constructed by using an SPR disk modified with in situ synthesized AgNPs (Figure 11b). let7a, whose dysregulation results in aberrant cell differentiation leading to the insurgence of cancer, plays a significant prognostic role. Using the hybridization chain reaction (HCR) strategy, a visible change in the SPR angle was observed when the target was present. The increase of SPR angle once the target was captured, was improved due to the in situ synthesis of AgNPs that were intercalated in the dsDNA resulting from HCR. The proposed biosensor reached a limit of detection of 0.35 fM [88] (Table 1). AgNPs can also be found in combination with AuNPs, as it was proposed in a study carried out by Liu’s research group [155]. A SPR sensor was constructed by using an Au film where the stem-loop capture DNA probe was immobilized. The stem-loop structure unfolds when target miR-21 is present. AuNPs functionalized with DNA start forming a super-sandwich complex on the unfolding structure. Then, AgNPs are added to this super-complex resulting in a further shift of the SPR angle. The sensitivity that they obtained was 0.6 fM (Table 3).

#### 7.1.3. Other Metallic Nanoparticles

A fluorescent sensor was constructed by combing a Rolling cycle amplification approach and in situ synthesis of copper NPs (CuNPs). let-7a was detected by an ssDNA padlock probe (Figure 11c). Once recognized, the resulting poly-T ssDNA was used as a template for the formation of fluorescent CuNPs, whose intense emission eliminated background signals showing a great degree of sensitivity (LOD of 70.6 fM) [89]. Magnetite (Fe_3_O_4_) nanoparticles, associated with carbon (Fe_3_O_4_@C), were used as a quencher in a fluorescence-based detection of miR-20a. As soon as the catalytic hairpin assembly (CHA) indirectly covalently initiates in the presence of miR-20a on Fe_3_O_4_@C complex, the two DNA hairpins H3 and H2, associated with Fe_3_O_4_@C originally bond, detach, and the fluorescence of H2 cannot be quenched by Fe_3_O_4_@C nanoparticles. The fluorescence signal generates results that are proportional to miR-20a concentration, reaching an LOD of 491 fM [90] (Table 1). 

### 7.2. Graphene Oxide

**Figure 12:**
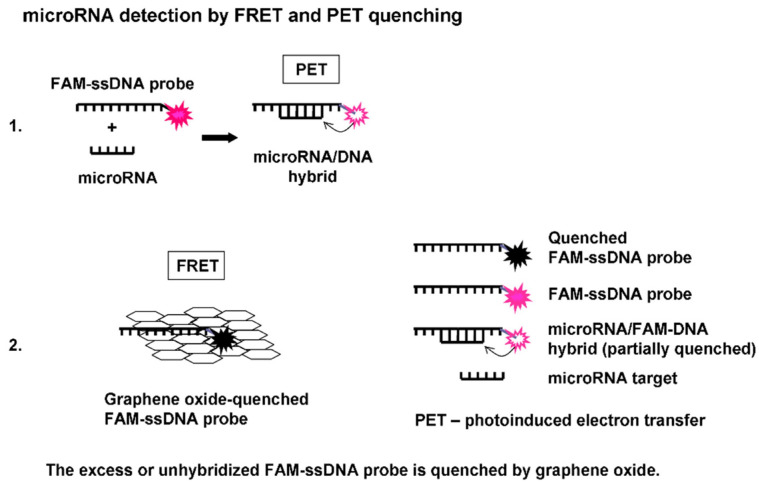
Graphene oxide FAM-ssDNA quenching mechanism for miR-21 optical detection strategy. Adapted with permission from Ref. [92]. Copyright (2021). MDPI Open access article under the Creative Commons Attribution License.

As a carbon-based nanomaterial, the size of the sp^2^ and sp^3^ configurations determine the GO band gap. Indeed, GO is characterized by a large band gap responsible for broad-band fluorescence (near-infrared, visible, and ultraviolet regions). GO has two known fluorescence bands: one at 430 nm and another at 550–600 nm. GO also acts as a quencher due to their electron transfer or Foster resonance electron transfer [162,163]. Nitu and coworkers exploited GO quenching activity proposing a double fluorescence quenching approach for an optical biosensor aimed to detect miR-21. In this study, the first quenching method consists of the recognition of the target by a fluorescent-labeled ssDNA probe (FAM-ssDNA), which is quenched upon binding via a photo-induced electron transfer (PET). Then, GO is added and it binds and quenches the unhybridized FAM-ssDNA, making possible the quantification of target concentration (Figure 12) [92] (Table 1). The same target (miR-21), but a different working principle was illustrated by Shin et al. where GO was used as a substrate for the covalent functionalization of a fluorescent dsDNA probe, associated with locked nucleic acid (LNA). When the target binds the probe in the complementary region, the fluorescent strands are released. The role of GO was important for improving fluorescence recovery [156].

### 7.3. Quantum Dots

**Figure 13:**
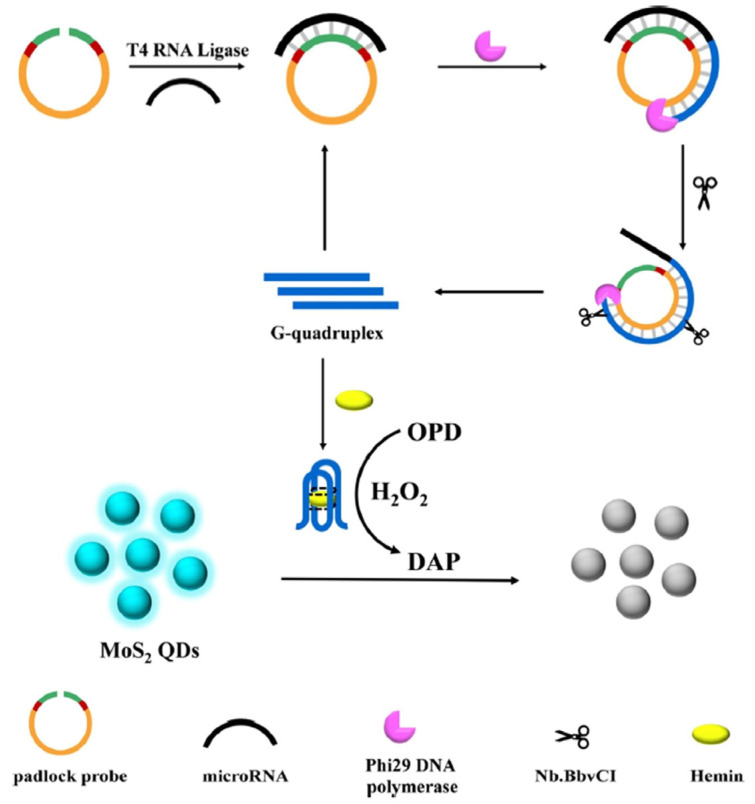
let7a detection via RCA causing suppression of MoS_2_ QDs fluorescence due to DAP enzyme, produced by the conversion of G-quadruplex- HRP activity after the interaction with hemin. Adapted with permission from Ref. [154]. Copyright (2020). American Chemical Society.

QDs are characterized by a wide excitation and narrow emission, with high sensitivity and specificity in optical sensing for biological applications. QDs optical features are tunable with their size. To better understand QDs properties, it is important to mention the “trap states” energies. In general, an electron moving from the valence band to the conductive band can leave an “hole”. The resulting electron hole may be trapped by low energy states, known as “trap states” and cannot be recombined for generating heat or light. To solve this issue, it is possible to “fill” the holes by adopting a surface passivation strategy. The latter consists of the employment of an energy bandgap semiconductor (e.g., CdSe passivated with ZnS). For all the abovementioned properties, QDs can be used as fluorescent dyes since they reach quantum yields similar to the ones of organic dyes, but also as fluorescent probes [94] (Table 1). Fluorescent molybdenum disulfide (MoS_2_) QDs, without any chemical modification, were used for let-7a quantification in human serum (Figure 13). The mechanism consisted of the initiation of rolling amplification cycles after the encounter with target miRNA, resulting in G-quadruplex DNAzyme, bearing horseradish peroxidase activity (HRP). These latter, in the presence of hemin, were responsible for the production of 2,4-diaminophenazine (DAP) able, by chance, to suppress the fluorescence of MoS_2_ QDs via an inner filter effect (IFE). The limit of detection was 4.6 fM [154]. In another work, miR-155 was detected by using two different color-emitting CdTe QDs. When green-fluorescent emitting QDs were in presence of the dsDNA probe, they did not experience any fluorescence change and, after their addition, neither did orange-fluorescent CdTe QDs. However, miR-155 capturing caused green-emitting QDs to aggregate and their fluorescence was quenched, displaying an “off-state”. After target recognition by green-QDs, orange-QDs were added and did not shift to “off-state” but their fluorescence appeared to increase as a result of a short distance allowing the fluorescence resonance energy transfer (FRET). The detection limit achieved using this strategy was of 14.0 pM [152]. 

### 7.4. Nanostructured Polymers

**Figure 14:**
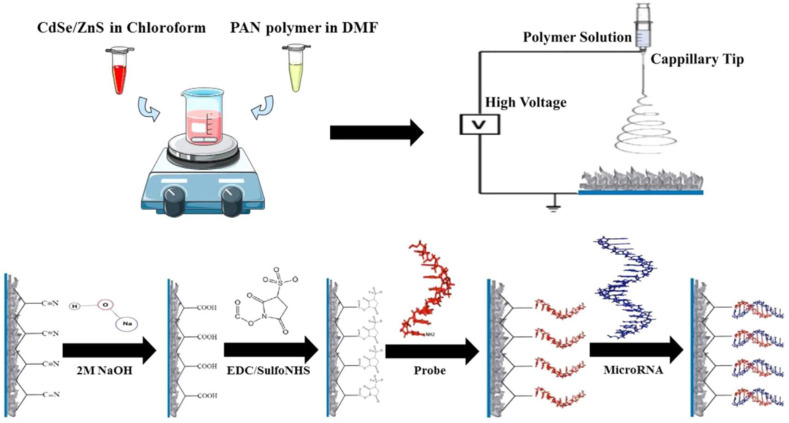
Schematic representation of fluorescent polyacrylonitrile (FPAN) nanofiber production (via electrospinning) and subsequent surface functionalization with complementary probe for miR-21 detection. Adapted with permission from [157]. Copyright (2020). The Author(s). Published by IOP Publishing Ltd. Open access article under the Creative Commons Attribution License.

Nanostructured polymers exhibit numerous optical properties such as photoluminescence, electrochemiluminescence, and nonlinear optical properties. Each polymer, due to its own structural patterns and matrices, can scatter the light and also allow the immobilization of species bearing optical properties as well (e.g., MNPs, crystals, dyes, bioluminescent molecules) [97] (Table 1). There are two examples of this latter combination polymer-optical active species that are provided in the following text as an alternative strategy for miRNA optical sensing. Fluorescent polyacrylonitile (FPAN) nanofibers associated with CdSe/ZnS QDs, were produced via an electrospinning technique to obtain a substrate for a fluorescent sensor aimed to detect miR-21 (Figure 14). The great advantage was provided by the large surface area–volume allowing the immobilization of the DNA detection probe. This feature increased the possibility to capture the target miRNA, making this approach a successful strategy. FPAN induced a sensitivity of 1 pM [157]. Polydiacetylene (PDA) is an interesting material employed for its optical properties, such as color and fluorescence. Zhu and colleagues proposed a PDA-based microtube optical sensor functionalized with gold nanorods (PDA@AuNRs) for the detection miR-21. Using a thiol-modified DNA probe, the system was able to hybridize the analyte of interest whose concentration was proportional to the fluorescence signal measured at the tip of the microtube, after irradiating at 532 nm excitation light. The PDA@AuNRs were demonstrated to be sensitive and selective sensors showing an LOD of 0.01 nM [158]. 

## 8. Concluding Remarks

In recent decades, miRNAs have consolidated their role as cancer biomarkers. For years, the old standard techniques were considered the best strategies for detecting miRNA. They involved several steps of sample preparation, with RNA extraction as the starting point. Due to their short sequences, these old techniques rely on amplification strategies (such as RT-qPCR), hybridization with complementary probes (e.g., Northern blot, microarrays), or sequencing (RNA-seq). Although these techniques represent the basic strategy for miRNA detection, they present several criticisms. First of all, these approaches require several preparatory steps, which happen to require specialized personnel, precise instruments, and disposable materials to preserve the samples and avoid contamination. Another crucial point is the lack of a transducer element. The need to overcome the main limitations of standard molecular techniques (high cost, long sample preparation, time-consuming steps, and a lack of transducer elements) and the emerging advantages of nanotechnology are paving the way for the use of nanostructured biosensors as alternative methods for miRNA detection. In this review, nanostructured biosensors are reported as valid tools for miRNA detection. In general, biosensors are defined as devices consisting of a detection system and a transducer element that provide analytical information. Due to the low concentration of miRNA in body fluids (attomolar/femtomolar concentrations), biosensors can be combined with amplification techniques that allow elongation of short miRNA sequences but also increase the probability of target binding. Nanostructured electrochemical and optical biosensors have remarkable advantages, such as a large surface area–volume ratio, the possibility to immobilize custom probes on their surface, and the ability to convert a biorecognition event into a measurable signal. All these aspects provide high sensitivity and specificity in detecting miRNAs compared to the old standard techniques. Regarding the fabrication of a nanostructured biosensor, the need to select a certain type of nanomaterial over another is based on the goal that researchers want to reach. In this work, four nanomaterials have been proposed as suitable tools for both electrochemical and optical approaches. Metallic nanoparticles (MNPs) possess conductive features but also optical characteristics, such as localized surface plasmon resonance (LSPR), that turn them into valid substrates for both types of the overmentioned biosensors. Most of graphene oxide’s (GO) properties rely on its peculiar chemical structure exposing multiple functional groups (carboxyl, epoxy, and hydroxyl group). GO chemical configuration leads to notable electrical, electrochemical properties, but also to its structure-dependent absorption spectra and Raman fingerprint. In addition, GO is exploited for the immobilization of complementary probes to target miRNA, but also as a scaffold for the functionalization of other nanomaterials. On the other hand, quantum dots (QDs) are semiconductor nanoparticles that are appropriate as electrode modifiers but have been mainly exploited for optical sensors since they show intrinsic photoluminescence making them optical signal amplifiers. Nanostructured polymers present several properties that depend on the type of polymeric units implemented during their fabrication process. For instance, some polymers possess natural fluorescence properties that can fit with an optical application or can show a degree of electrical conductivity for electrochemical implementation. 

Regardless of the type of nanomaterial employed and the transduced signal, nanostructured biosensors offer the possibility to manage a small quantity of sample due to the fact that they can integrate other technologies, such as microfluidic systems, making them appropriate for miRNA detection since they are present in several body fluids (blood, plasma, saliva, and so on).

Future prospects aim at optimizing the fabrication of nanostructured biosensors to achieve high sensitivity lowering the detection limit (LOD), thus overcoming all the limitations of the old standard methods for miRNA detection.

## Figures and Tables

**Table 2:** Electrochemical biosensors for miRNA detection.

Target	Electrochemical Signal	Nanomaterials	Detection Strategy	LOD	Ref.
**miR-21**	DPV	AuNPs	Hybridization with capture miRNA probe	1 fM	[132]
	SWV	Biotinylated-AgNPs, Streptavidin-AgNPs	SDR amplification	0.4 fM	[84]
	DPV	AuPtBNPs	Hybridization with cDNA capture probe	0.63 fM	[86]
	ECL, EIS	CQDs-Au-PEI@TiO_2_	CHA amplification	0.03 fM	[133]
**miR-21-5p**	EIS	AuNPs	Hybridization with anti-miRNA probe	4.31 fM	[83]
**miR-155**	DPV	AgNPs	DNA walker amplification	3.2 fM	[134]
	DPV	3D-N-doped rGO/AuNPs	Hybridization with AuAgNR/Thi/F complementary DNA probe	1 pM	[135]
**miR-222**	DPV	rGO, AuNPs, DNA-CuNPs as signal probe	HCR amplification	0.03 fM	[136]
**miR-24**	DPV	AuNPs/PEDOT	Hybridization with DNA capture probe	0.38 fM	[137]
	DPV	PANI/PA	Hybridization with DNA capture probe	0.34 fM	[138]
**Multiplex detection: miR-155 and miR-21**	DPV	AuNPs/rGO, AuNPs/MoS_s_	Hybridization with DNA capture probe	For AuNPs/RGO 12.0 nM (miR-21) 25.7 nM (miR-155) For AuNPs/MoS2 51.6 nM (miR-21) 59.6 nM (miR-155)	[139]
**Multiplex detection: miR-155, miR-21 and miR-210**	SWV	AuNPs/GQDs/GO	Hybridization with miRNA probe	0.04 fM (miR-21) 0.33 fM (miR-155) 0.28 fM (miR-210)	[140]

**Table 3:** Optical biosensors for miRNA detection.

miRNA Target	Optical Signal	Nanomaterial	Detection Strategy	LOD	Ref.
miR-155	Colorimetric assay	C-AuNPs and P-AuNPs	Hybridization with hairpin-DNA probe	100 aM	[151]
	Fluorescence	Green and orange-emitting CdTe QDs	Hybridization with ds-DNA probe	14 pM	[152]
miR-141	SERS	Au@MNPs	Hybridization with labeled (Raman reporter) DNA probe	100 fM	[153]
let-7a	SPR	AgNPs	HCR	0.35 fM	[88]
	Fluorescence	CuNPs	RCA	70.6 fM	[89]
	Chemiluminescence	MoS_2_ QDs	RCA	4.6 fM	[154]
miR-21	SPR	AuNPs@DNA@AgNPs	Hybridization with AuNPs@DNA supersandwich structure	0.6 fM	[155]
	Fluorescence	GO	Hybridization with fluorescent-labeled ssDNA probe	1 nM	[92]
	Fluorescence	GO	Hybridization with fluorescent-labeled DNA probe	99.3 pM	[156]
	Fluorescence	FPAN@CdSe/ZnS QDs	Hybridization with miRNA-21 complementary probe	1 pM	[157]
	Fluorescence	PDA@AuNRs	Hybridization with DNA probe	0.01 nM	[158]
miR-20a	Fluorescence	Fe_3_O_4_@C	CHA	4.91 × 10^−4^ nM	[90]

## Data Availability

This Review doesn’t include original scientific data beyond those already published.
